# Questionnaires for Lung Health in Africa across the Life Course

**DOI:** 10.3390/ijerph15081615

**Published:** 2018-07-31

**Authors:** Sepeedeh Saleh, Richard van Zyl-Smit, Brian Allwood, Herve Lawin, Bertrand Hugo Mbatchou Ngahane, Irene Ayakaka, Elvis Moyo, Asma El-Sony, Kevin Mortimer

**Affiliations:** 1 Liverpool School of Tropical Medicine, Liverpool L3 5QA, UK; sepeedeh.saleh@lstmed.ac.uk (S.S.); jamie.rylance@lstmed.ac.uk (J.R.); kevin.mortimer@lstmed.ac.uk (K.M.); 2 Division of Pulmonology, University of Cape Town, Rondebosch 7701, South Africa; richard.vanzyl-smit@uct.ac.za (R.v.Z.S.); brianallwood@gmail.com (B.A.); 3 University of Cape Town Lung Institute, Cape Town 7701, South Africa; richard.vanzyl-smit@uct.ac.za (R.Z.S.); brianallwood@gmail.com (B.A.); 4 Unit of Teaching and Research in Occupational Health and Environment, University of Abomey-Calavi, Calavi, Cotonou 01 BP 526, Benin; hervelawin@gmail.com (H.L.); 5 Douala General Hospital, Department of Internal Medicine, Douala, Cameroon; mbatchou.ngahane@yahoo.com (B.H.M.N.); 6 Makerere University College of Health sciences; Uganda Tuberculosis implementation Research Consortium (U-TIRC), Kampala 7072, Uganda; ayakaka@gmail.com (I.A.); 7 Malawi Liverpool Wellcome Trust Clinical Research Programme, Queen Elizabeth Central Hospital, College of Medicine, Chichiri 30096, Blantyre 3, Malawi; emoyo@mlw.mw (E.M.); jamie.rylance@lstmed.ac.uk (J.R.); 8 Epidemiological Labarotory (EpiLab) for Public Health Khartoum 3, Block 3- Building 11, Khartoum, Sudan; asmaelsony@gmail.com (A.E.S.)

**Keywords:** lung heath, symptoms, air pollution, tuberculosis, COPD, questionnaires, cross-cultural adaptation

## Abstract

Respiratory infections remain a leading cause of morbidity and mortality in many low and middle-income countries but non-communicable disease rates are rising fast. Prevalence studies have been primarily symptom-focused, with tools developed in countries in the Global North such as the United States and the United Kingdom. Systematic study in sub-Saharan African populations is necessary to accurately reflect disease risk factors present in these populations. We present tools for such studies, developed as part of the International Multidisciplinary Programme to Address Lung Health and TB in Africa (‘IMPALA’), which includes lay representatives. At a preliminary meeting, the adequacy and suitability of existing tools was discussed and a new questionnaire set proposed. Individual questionnaires were developed, and an expert panel considered content and criterion validity. Questionnaires underwent a cross-cultural adaptation process, incorporating translation and contextual ‘sense-checking’, through the use of pre-established lay focus groups in Malawi, before consensus-approval by project collaborators. The complete set of research questionnaires, providing information on lung health symptoms and a relevant range of potential risk factors for lung disease, is now available online. In developing the tools, cultural and contextual insights were important, as were translational considerations. The process benefitted from a foundation in expert knowledge, starting with validated tools and internationally respected research groups, and from a coordinated collaborative approach. We present and discuss a newly devised, contextually appropriate set of questionnaires for non-communicable lung disease research in Africa that are now available in open access for all to use.

## 1. Introduction

Lung disease is a leading contributor to morbidity and mortality in sub Saharan Africa [1,2]. Whilst respiratory infections such as tuberculosis (TB) are still a leading cause of morbidity and mortality in this region, their relative disease burdens are decreasing, with concurrent increases in non-communicable disease rates [3]. Data on the prevalence of non-communicable lung disease in the sub Saharan African region are limited, with underdiagnosis in many areas a key factor [1]. The available data show wide variations in both chronic obstructive pulmonary disease (COPD) and asthma prevalence in sub Saharan Africa. A recent systematic review reported COPD prevalences between 4·1% and 24.8% depending on the diagnostic methods used [4]; and findings from a leading international study of asthma across sub Saharan Africa (The International Study of Asthma and Allergies in Childhood, or ISAAC) show differences in prevalence from 9.1% in Ethiopia, to 20.3% in South Africa, with higher prevalences reported in urbanised areas [5].

Drivers of these non-communicable lung diseases in sub Saharan Africa include poor indoor air quality (e.g., combustion of biomass fuel for lighting and cooking), outdoor air pollution (e.g., from motor vehicles), tobacco smoking, undernutrition [6] and high rates of acute infection [7]. Rapid urbanisation in countries across the region [8], and subsequent shifts in these environmental exposures at home and at work, will lead to changing patterns of chronic lung pathology [5].

Multinational projects such as the Burden of Obstructive Lung Disease (BOLD) study [9], the Global Asthma Network (GAN) and the International Union Against Tuberculosis and Lung Disease have provided information about the global prevalence, drivers, and outcomes of respiratory disease. However, the changing epidemiology of non-communicable disease in low and middle-income countries demands further systematic study [1,8]. Prevalence studies have been primarily symptom-focussed, using tools developed in the Global North. To ensure these accurately reflect the breadth of disease risk factors to which the poorest populations are exposed, measures of assessment specific to African populations are necessary.

We have co-devised tools with improved relevance to sub-Saharan Africa as part of a collaborative project aiming to develop methodologies for measuring non-communicable lung disease exposures and outcomes, and supporting and facilitating research in resource-constrained African environments. This has involved the creation of a strategic multi-disciplinary partnership of paediatric and adult lung health investigators from Benin, Cameroon, Ethiopia, Ghana, Kenya, Malawi, Nigeria, South Africa, Sudan, Tanzania and Uganda, with underpinning support from the Pan African Thoracic Society (PATS), American Thoracic Society (ATS) Methods in Epidemiologic, Clinical and Operations Research (MECOR) programme, MRC BREATHE-Africa partnership, Malawi Liverpool Wellcome Trust (MLW), Burden of Obstructive Lung Disease (BOLD) Centre, Global Asthma Network (GAN), and Collaboration for Applied Health Research and Delivery (CAHRD). Members of this unique partnership worked with lay in-country representatives, developing context-sensitive tools, initially for Malawi [10]. In Malawi and beyond, a comprehensive set of questionnaires will support the mapping, tracking and interventions required to maximise health in populations affected by the double burden of infectious and chronic non-communicable diseases 

## 2. Methods

At a preliminary meeting of the ‘IMPALA’ International Partnership (www.lstmed.ac.uk/impala), representing expertise in asthma, COPD, air quality, smoking and occupational lung disease, we identified existing validated tools, including the World Health Organization household energy use survey and questions on nutrition developed by experts from the Global Asthma Network. After consultation with the original authors and developers, we held structured discussions with in-country colleagues to identify areas in which existing questionnaires were inadequate, improperly focussed or unsuitable for direct deployment in sub Saharan Africa. The agreed targets were context-appropriate tools to assess lung disease symptoms, smoking, nutrition, household energy use, history of tuberculosis, and wider lifetime exposures to risk factors for lung disease, including occupation (see Table 1).

New questionnaires were collaboratively created on a shared online platform (Kobo toolbox). Where possible we adapted existing tools: others were developed *de novo*. Insights from external advisors with specific expertise (e.g., dietary contributors to lung health) and situational knowledge relating to the Malawian context were sought and used to refine questionnaire scope and design. A single co-ordinating team ensured coherence and completeness across the tools with collaborative meetings, both remotely and in person, held throughout the process to share insights and progress updates. Prior to any further processes, the questionnaires’ content validity (the extent to which the tool measures the complete spectrum of the construct) and criterion validity (in comparison to a ‘gold standard’ or proxy) were considered by an expert panel with broad previous field research experience. The questionnaires were translated into Chichewa by two Malawian translators, with two independent parallel translations in most cases. Subsequent discussions were held between translators and a research coordinator to resolve areas of difficulty or discord.

Original and translated versions were then taken forward for consultation in a series of meetings with pre-established lay focus groups, locally known as community advisory groups [11]. Separate meetings, each of approximately nine people from urban Blantyre and from rural Chikwawa, gave feedback on the comprehensibility and contextual relevance of the questionnaires, within a pre-agreed consultation framework. Meetings were conducted in a combination of English and Chichewa, with fieldworkers and community advisory group coordinators in key positions as linguistic intermediaries. Lay members were organised into subgroups of three, each with a bilingual member of staff with prior understanding of the questionnaire content in a coordinating position. This allowed for guided debate where all voices could be heard, before consensus opinion was sought amongst the wider group. In terms of participant selection, purposive sampling was used to allow fuller representation by age, gender and occupation.

The consultation meetings were part of ongoing work within the science communication group, with specific Research Ethics Committee approval granted for the testing of the questionnaires in Malawi (College of Medicine Research Ethics Committee P.02/18/2349) and planned for other sites.

Questionnaires with more original content and those with significant anticipated context-sensitive elements were fully considered by focus groups from both urban and rural areas. Others, based on established sources were only put to one group, although members considered how the questions might be understood and applicable more broadly (detailed in Table 1).

The resulting questionnaires, including flow and skip-logic were circulated, first to key researchers in the team, then to the wider expert group for feedback. Suggested changes were discussed and agreed on amongst coordinators before incorporation to the final questionnaire set. Partners responsible for each individual tool also nominated key questions to be taken forward to form a composite ‘screening questionnaire’. The new questionnaires are being used by members of the wider collaboration in pilot projects, initially in Malawi and subsequently, after iterative adaptation processes, in other sites across sub Saharan Africa.

## 3. Results

Through a rigorous process of questionnaire development and cross-cultural adaptation we have devised a novel set of questionnaires aimed at measuring chronic lung disease outcomes and a relevant range of potential risk factors in the sub Saharan African context (Table 1). 

Wide consultation was employed throughout the development process, both in terms of geography and discipline, and mixed-methods maximised relevance across the board. In addition to the original partnership, external experts were consulted for various aspects of specific questionnaires, including expertise in nutritional aspects and assessment of air pollution.

In translating the tools prior to in-country consultation, we found that a structured sequential approach, akin to an adapted ‘dual panel approach [10], helped to minimise development time whilst conserving important elements of the process. Subsequent focused discussions between research coordinators and translators helped to maintain communication and consensus throughout the wider team: key themes throughout all phases of the process.

Consultation group members were adults, with an approximately equal mix of males and females aged between 25 and 40. A balance between continuity and fresh perspective was found to be important. Through successive meetings, a minority of members were maintained, with new participants co-opted to maximise the demographic mix. 

Initial division of the groups into three with an assistant guiding each subgroup allowed the research coordinator, with more rudimentary Chichewa, to probe key areas previously identified by the researchers, to unpick the reasoning behind suggested changes, and to resolve disparities of opinion. Debrief sessions promoted iterative learning across multiple sessions.

Contextual insights, particularly relating to activities of daily living, emerged throughout the process of devising the questionnaires and their subsequent translation and cross-cultural adaptation.

The translation process revealed underlying categorisation problems, for instance in the case of COPD and asthma, which could not be simply translated, and for which suitable descriptions were the source of extensive discussion. Similarly, the requirement became apparent for separate words or phrases to distinguish daily “activity” from “exercise purely for recreation”, to capture both adequately. Numerous changes were made by focus group members to reflect locally spoken forms of the language (rather than the formal translations provided by interpreters), for example in the commonly used words for gaining and losing weight. These areas will be revisited in the ensuing translation and validation processes in other sub Saharan Africa countries.

In terms of cross-cultural adaptation, further contextual insights became apparent. First, the development team had to consider the dichotomy between farming as an occupation and subsistence farming as a daily activity carried out by most Malawians, ensuring that this was appropriately reflected across the questionnaire set. This was a fundamental departure from the original questionnaires, aimed at lifestyles of people living in countries of the Global North. In terms of nutrition, also, significant changes had to be made to previous tools, making provision for important Malawian food and drink categories, including nsima (a maize-based staple), insects and frogs, and locally produced alcoholic and non-alcoholic drinks.

Areas of the questionnaires relating to exposure to potential respiratory insults, such as from farming or livestock, again involved significant changes, and varied between urban and rural groups. Questions exploring road-derived particulates originally differentiated between ‘dust’ and ‘tarmac’ surfaces as a proxy for traffic volumes, but the consideration of prominent major dust roads in Malawi led to the use of the categories ‘main’ and ‘minor’ instead, with road surfaces captured only to indicate the type of particulate exposure.

This first questionnaire set is currently available electronically on an online platform for formal validation and piloting by our collaborators. This will allow development of further context-appropriate tools across the region, with the in-depth cross-cultural adaptation (CCA) process forming a platform for efforts in adapting the tools for other populations.

## 4. Discussion

The development processes described above, in keeping with the literature on questionnaire design, validation, and cross-cultural adaptation [10,13] have allowed for the integration of contextual factors specific to sub Saharan Africa, providing a novel set of tools for the assessment of chronic lung disease and its risk factors.

How well assessment tools measure endpoints in theory (approximating to content validity) and in reality (approximating to construct validity) are significantly affected by context and language. This makes the cross-cultural adaptation process (CCA), including translation, critical to their development. In this study, insights into daily life including indoor and outdoor environments, food and drink, and linguistic factors, as described above, have all contributed to improved questionnaire validity.

In terms of language in particular, whilst forward-backward translation has been widely used to ensure language fidelity [14], broader consultation with bilingual experts can be more effective [15]. We used both, starting with a dedicated translation service in Malawi for English-Chichewa translation, with two independent parallel translations in most cases, and structured discussion of particular areas of difficulty involving the research coordinators. This process helped to ensure that translations appeared clear, rational, and faithful to the clinical purpose (face validity).

Strengths of the process were its basis in expert knowledge, starting with respected, validated tools and with input from internationally respected research groups; and its collaborative nature throughout. This distributed leadership allowed the combination of global and local viewpoints, and “expert” and lay perspectives to be synthesised. Words, phrases and expressions were questioned and debated at each stage and remained relevant to the content of the tools in a way which we had not previously imagined. We are collating the linguistic and contextual knowledge amassed from this experience to allow its application in future projects. A specialist/medical “English-Chichewa glossary” is being developed which will ensure that efforts are not duplicated, and this work can contribute to future lung health research. In negotiating these contextual aspects, access to two different translators, local staff, and focus group members, all prepared to engage in repeated discussions of language and related context, proved invaluable. Central management was essential here to effectively co-ordinate activities and broker consensus. 

Recognising the potential for established community engagement structures [11] to skew the focus of community feedback, we mitigated this by frequent, open discussions between stakeholders to reconcile ideas and set shared agendas and action plans.

The process of cross-cultural adaptation and validation, as described above, entails significant resources in terms of time and staff commitment. Whilst development of the current questionnaires has contributed to the groundwork for further adaptations of these tools across the wider region, development of these additional versions, if done to the necessary standards, will demand further resources, and therefore constitute a not-insignificant undertaking.

The questionnaires are now freely available through the International Multidisciplinary Programme to Address Lung Health and TB in Africa (IMPALA) website, and we encourage other researchers to use them in order to capture and record lung health data in a standardised manner across the region.

Next steps in the process include collection of air quality data through particulate exposure monitoring, which is currently underway in the areas for which these tools were developed. Correlation of interview responses with these data will allow further validation of the tools. In reality, the finer aspects of validation and data collection are, to an extent, iterative, with incorporation of qualitative and quantitative data from partners in countries across sub Saharan Africa contributing to the development and improvement of further versions of the tools.

## 5. Conclusions

Recognising the need for context-informed lung disease tools, we developed an international partnership of lung health researchers and devised a questionnaire set to measure non-communicable lung disease exposures and outcomes, with improved relevance for Sub Saharan Africa. The development of these tools involved a process of cross-cultural adaptation, with collaboration between international lung health experts, local research teams and lay consultation groups. The questionnaires are now available on an online platform for further adaptation and use by research teams across the region. 

## Figures and Tables

**Table 1:** Questionnaires included in the final toolbox.

Questionnaire	Information Included	Focus Group(s) Consulted
Respiratory symptoms	Cough, wheeze, breathlessness, other symptoms; previous respiratory diagnoses; limitation in activities of daily life (including physical and psychological aspects)	Urban & Rural
Smoking	Current and life history of smoking tobacco, cannabis, waterpipe, e-cigarettes, and smoking of other substances	Urban only
Nutrition	Screening questions on amount of food eaten (per day) and questions on frequency of consumption of foods from main food groups, including drinks (alcoholic and non-alcoholic)	Urban & Rural
Household energy use	Devices and energy sources used for cooking; heating and lighting (including frequency and duration of use). Also incorporates questions on ventilation	Urban & Rural
History of TB	History of previous tuberculosis (TB) diagnoses and treatments, to inform about ‘Tuberculosis associated chronic obstructive pulmonary disease’: TOPD [12]	Urban only
Wider lifetime exposures	Air pollution exposures at home not previously covered and those outside the home. Includes exposure to passive smoking, outdoor fires, traffic fumes/dust, occupational exposures, and animal sources.	Urban & Rural
